# Organizational characteristics of highly specialized units for people with dementia and severe challenging behavior

**DOI:** 10.1186/s12877-024-05257-x

**Published:** 2024-08-14

**Authors:** Gerrie van Voorden, Raymond T. C. M. Koopmans, Mijke M. Strik-Lips, Martin Smalbrugge, Sytse U. Zuidema, Anne M. A. van den Brink, Anke Persoon, Richard C. Oude Voshaar, Debby L. Gerritsen

**Affiliations:** 1 Radboudumc Research Institute for Medical Innovation, Radboud University Medical Center, Nijmegen, Netherlands; 2 Department of Primary and Community Care, Radboud University Medical Center, Nijmegen, Netherlands; 3 University Knowledge Network for Older Adult Care Nijmegen (UKON), Radboud University Medical Center, Nijmegen, Netherlands; 4 Radboudumc Alzheimer Center, Radboud University Medical Center, Nijmegen, Netherlands; 5 Joachim en Anna, Center for Specialized Geriatric Care, De Waalboog, Nijmegen, Netherlands; 6 Department of Medicine for Older People, Amsterdam UMC, Location Vrije Universiteit Amsterdam, Amsterdam, Netherlands; 7 Amsterdam Public Health Research Institute, Aging and Later Life, Amsterdam, Netherlands; 8 Department of Primary and Long-Term Care, University of Groningen, University Medical Center Groningen, Groningen, Netherlands; 9 Alzheimer Center Groningen, Groningen, Netherlands; 10 Department of Psychiatry, University of Groningen, University Medical Center Groningen, Groningen, Netherlands

**Keywords:** Challenging behavior, Long-term care, Dementia, Treatment, Organizational, Physical environment, Sensory stimuli

## Abstract

**Background:**

People with dementia and severe challenging behavior in the Netherlands can be temporarily admitted to highly specialized units when their behavior is not manageable in regular dementia special care units (DSCUs). With scarce evidence available for the treatment of these patients, treatment in these units is in a pioneering phase. To gain more insight into these units, this study investigated organizational characteristics, i.e. admission and discharge characteristics, staffing, the physical environment, and the management of severe challenging behavior.

**Methods:**

Three data collection methods were used: 1) a digital questionnaire to be completed by the unit manager, 2) an interview with the physician responsible for medical care and often another practitioner, and 3) an observation of the physical environment for which the OAZIS-dementia questionnaire was used. Descriptive analysis was used for quantitative data and thematic analysis for qualitative data, after which data was interpreted together. Thirteen units participated, with their sizes ranging from 10 to 28 places.

**Results:**

Patients were mainly admitted from regular DSCUs, home or mental health care, and discharged to regular DSCUs. A multidisciplinary team comprising at least an elderly care physician or geriatrician, psychologist, and nursing staff member and other therapists as needed provided the treatment. Nursing staff hours per patient considerably differed among units. Nursing staff played a central role in the treatment. Competences such as reflectiveness on one’s own behavior, and being able to cope with stressful situations were described as relevant for nursing staff. Investing in a stable nursing staff team was described as important. The units varied in whether their work-up was more intuitive or methodological. In the diagnostic phase, observation together with an extensive analysis of the patient’s biography was essential. The units used a broad variety of interventions, and all paid attention to sensory stimuli. In the observation of the physical environment, the safety scored well and domesticity relatively low.

**Conclusion:**

Highly specialized units show strong heterogeneity in organizational characteristics and management, which can be understood in the light of the pioneering phase. Despite this, similarities were found in nursing staff roles, frequent multidisciplinary evaluation, and attention to sensory stimuli.

**Supplementary Information:**

The online version contains supplementary material available at 10.1186/s12877-024-05257-x.

## Background

Challenging behavior in persons with dementia – also known as behavioral and psychological symptoms of dementia (BPSD) or neuropsychiatric symptoms [1] – is common in nursing homes, with a mean prevalence of 82% [2]. The burden of challenging behavior is high, being associated with a lower quality of life [3–5], and increased distress in caregivers [5–8]. Severe challenging behavior – especially aggression and agitation – is known to lead to admission to psychiatric services, specialist care units or long-term care [9]. Moreover, the costs of especially agitation at the end of life in dementia increases informal and formal health care costs by 30% [10]. A small proportion of people with dementia show very frequent or severe agitation with, a prevalence of 7.4% and 6.3%, respectively [11, 12]. For very frequent physical aggression and very frequent vocalizations, a two-week prevalence of 2.2% and 11.5%, respectively, has been found in nursing home patients [13].

In the Netherlands, most people with dementia who cannot live on their own anymore live in a dementia special care unit (DSCU) [14]. Although DSCUs have varying characteristics, common elements are the psychogeriatric expertise of trained staff and activities that meet the needs of the people with dementia in a tailored environment [15, 16]. In the Netherlands, DSCUs commonly have a multidisciplinary team available that comprises of an elderly care physician, a health care psychologist, and nursing staff – the majority regards certified nursing assistants—which can be extended with therapists, i.e. physiotherapist, occupational therapist, speech therapist and dietician, when indicated [17–19]. Table 1 describes the roles and education of the usual team members in further detail.

**Table 1:** Role and education of elderly care physicians, health care psychologists and nursing staff in the Netherlands

***Elderly care physicians***
**Elderly care physicians** are medical doctors who have completed a three-year specialist training program in elderly care medicine where they worked 80% of their training time in clinical practice, and engaged one day a week in a training program at the university department. During their training time in practice, they work at least in a nursing home, a rehabilitation unit, a hospital department, and a geriatric psychiatry institution. Further specialized training is possible in primary health care, psychogeriatric medicine, geriatric rehabilitation, and palliative medicine [18, 20].
***Health care psychologists***
**Health care psychologists** are trained in a two-year post-master study program comprising an academic course and training in professional practice. During this training, they study diagnosis, care needs assessment, treatment and other duties. Training is provided by independent institutions throughout the Netherlands that work in close cooperation with Dutch universities. Health care psychologists often work in mental health, nursing homes or a general hospital [21].
***Nursing staff***
The **nursing staff** comprises **baccalaureate-educated registered nurses**, **registered vocationally trained nurses, certified nurse assistants**, **nurse assistants, and nurse aides** corresponding with qualification levels 6, 4, 3, 2 and 1 of the European Qualification Framework (EQF), respectively [17, 22]. **Baccalaureate-educated registered nurses** had graduated from a four-year training course at a university of applied sciences [17]. **Registered vocationally trained nurses** followed a four-year vocational education training course in a vocational education and training college. Both baccalaureate-educated registered nurses and registered vocationally trained nurses have a nationally qualified title, title protection and nurses are recorded in a national qualification register [17, 23]. **Certified nurse assistants** in the Netherlands are vocationally trained in an vocational education and training college in a two- to three-year training program, **nurse assistants** are trained in a two-year training program, and **nurse aides** in a half- to one year training program [17].

In the Netherlands, since approximately a decade, a small selection of people with severe challenging behavior can be temporarily admitted to highly specialized units when their behavior is not (regarded) manageable in a regular DSCU, such as behavior that causes serious safety problems, is very unpredictable or is very vocally disruptive. Several developments in health care have possibly contributed to the need for such units. First, the number of people with dementia is increasing whereas the number of nursing home places is not growing accordingly [24, 25]. Second, nursing homes tend to have more people admitted with challenging behavior which also is more severe [26, 27]. Third, admission possibilities in mental health care have been phased out in the Netherlands, leading to a decrease of 25.4% of admission days at wards in mental health care for people with delirium, dementia or other amnestic and cognitive disorders from 2012 to 2018 [28]. Fourth, it is believed that people with dementia and severe challenging behavior need expertise from both long-term care and mental health care [29]. Finally, the health care inspectorate has reported concerns about the quality of care for people with dementia and severe challenging behavior in the Netherlands [30]. These highly specialized units have been developed by long-term care organizations often in close cooperation with mental health care institutions.

However, at present little is known about these highly specialized units. For the aim of understanding whether these units contribute to a better quality of life and care for people with dementia and severe challenging behavior, it is needed to know what the organizational characteristics of these highly specialized units are. These insights can be used for further research into whether and why these units provide effective management of the challenging behavior. Elements of this knowledge about the management may eventually be proven useful in other settings. In Australia, a specialist residential dementia care program exists [31]. In one of these units, people with dementia and severe challenging behavior reside in an eight-place domestic-style residential cottage on average for twelve months, after which they are transferred to regular dementia care services [32]. Despite this example, little is known about this specific patient group, other similar care settings and the treatment applied there. Therefore, we studied these highly specialized units in the Netherlands to gain insights for clinical practice and further research.

Our aim was to describe the general characteristics of these units based on the following questions:

1. What are the organizational characteristics of these units regarding admission and discharge, staffing, and the physical environment?
2. What characterizes the management of severe challenging behavior on these units?

## Methods

### Sample and setting

Only units with temporarily admitted patients with dementia and severe challenging behavior in dementia were included. Units were identified and recruited by the six academic networks of long-term care [33]. At the start of this study, sixteen units were identified and invited to participate, fourteen of which gave consent. One of these units was closed at the start of the study, leaving thirteen units located throughout the Netherlands. Five units were part of a mental health care organization, and seven part of a long-term care organization, with two units in one organization (units 08 and 09), and one unit was a collaboration of both.

### Procedure

To answer the research questions, we used three data collection methods: 1) a digital questionnaire with mainly factual questions to be completed by the unit manager, 2) an interview about the treatment with the physician responsible for medical care, who was encouraged to invite another practitioner, and 3) an observation of the physical environment by the researcher. We chose these different methods to be able to answer our research questions, to provide for time for the unit manager to look up data, and to establish richer results for the topics competences of nursing staff, and physical environment. Data about these topics were integrated where applicable in the analysis [34]. Interviews and observations were scheduled on the same day and conducted at the workplace of the interviewees from May until August 2018.

### Measurements

#### Digital questionnaire

The digital questionnaire was self-developed with Lime Survey and sent to the unit managers [35]. The questionnaire comprised 43–48 mandatory, mainly fact-based questions at the unit level (see Table 2). Questions concerning the reasons for admission, competences and training of nursing staff, and work and education of the unit manager were open-ended. Twelve digital questionnaires were completed by the unit managers, and one by a baccalaureate-educated registered nurse in the unit due to time constraints of the unit manager. The patient administration had no exact data regarding residences before admission, number of compulsory admissions, discharge locations, and full-time equivalents of nursing staff (see Supplementary Materials 1–3), which were estimated by unit managers. Unit managers often had an educational background as baccalaureate-educated registered nurse after which they were trained in care management. They were on average 53.1 years old, and for 2.8 years involved.

**Table 2:** Items digital questionnaire for the unit managers

***Research questions:***	*Items:*
*1: Admission and discharge characteristics per unit*	- mean number of admissions per year - number of compulsory admissions as percentage at present - reasons for admission (open-ended) - mean age of admitted patients - residence before admission as percentage per year for given categories (home, DSCU, somatic care unit in a nursing home, hospital, mental health care institution, other) - mean length of stay in months - discharge location as percentage per year for given categories (home, back to referring unit, long-term care unit within the organization, long-term unit in another long term care organization, mental health unit for long term care, no discharge possible, other)
*1: Staffing*	- staff available in full time equivalents per education level (categories) - nursing staff hours from the working schedule per 24 h - sick leave nursing staff as percentage in 2017 (without maternity leave) - competences nursing staff (open-ended) - training nursing staff (open-ended)
*1: Physical environment*	- unit size as number of beds available
*2: Management of the behavior*	- use of guidelines (yes/no; if yes which) - use of clinical evaluation instruments (yes/no; if yes which)
***Details unit manager:***	- tenure in this unit since (year) - work experience (open-ended) - educational background (open-ended) - age

#### Interview

We developed an interview guide that followed the patient journey which was inspired by the (clinical) experience from the authors and piloted. It comprised of topics such as first day of admission, characterization of treatment, training in the management of behavior, and experienced difficulties (for all topics and questions see the interview guide in Supplementary Material 4). The interviews were conducted by the first author (GV), who was not known to the interviewees. The principal interviewee – the physician(s) responsible for medical care – was requested to invite another professional, preferably a psychologist as they are usually involved in case of challenging behavior on regular DSCUs [19]. All interviews were audiotaped and transcribed verbatim, and a summary of the transcript was sent to the interviewees as a member check. Twelve interviews were held, lasting between 56 and 85 min. The interviewees comprise nine elderly care physicians, two geriatricians, and one geriatric psychiatrist. In seven units, the (health care) psychologist joined, in one unit the other physician responsible for medical care, and in one unit the nurse practitioner. Interviewees where on average 46.4 years old, and 19% of the interviewees were male. Saturation was reached after nine interviews, in the sense that no new themes were identified.

#### Observation of physical environment

The OAZIS-dementia [36] was used, which has been developed to assess long-term care environments in a Dutch setting [37, 38]. The OAZIS-dementia has a good inter-rater reliability, with higher scores indicating a higher probability that the environment has positive effects on its residents [37]. It comprises 72 items to be rated on a five-point Likert scale, ranging from 1 ‘not at all’ to 5 ‘completely’ applicable. The instrument is divided into seven themes: 1) privacy and autonomy, 2) sensory stimulation, 3) view and nature, 4) facilities, 5) orientation and routing, 6) domesticity/small scale, and 7) safety. An example item from the theme facilities is: ‘there is enough space for the resident to receive visitors in his/her own room.’ In addition, we added items about the number of other rooms available and their function, e.g. the availability of a seclusion room. The OAZIS-dementia and general observation form was completed by GV. In two units, GV observed together with ML.

### Analysis

#### Quantitative data

The quantitative digital questionnaire responses and OAZIS-dementia scores were analyzed by the use of descriptive statistics. For each category in the OAZIS-dementia, the points reached were summed up and divided by the total number of items in this category. For the weighed final score, all items were summed up and divided by the total item number.

#### Qualitative data and data integration

Qualitative data from the digital questionnaire, interviews, and the observation of the physical environment was analyzed together. Investigator triangulation was realized by GV and ML jointly analyzing the interview transcripts supervised by DG, following the principles of thematic analysis [34]. GV and ML manually coded the first transcript separately by labeling meaningful fragments using open coding in a pragmatic way [39, 40], discussing differences until they reached agreement. Atlas.ti version 8.3.16 was used for coding [41]. The other interviews were coded by ML or GV and discussed. Codes referred to facts as well as experiences and views, in line with the interview questions asked. First, GV and ML analyzed coded text fragments that related to management of severe challenging behavior, which led to the merging and splitting of codes, finalized by a visualization of relevant themes in management according to the interviewees. Furthermore, remaining codes were analyzed together with the open-ended questions from the digital questionnaire about the competences of nursing staff and the description of the general impression of the physical environment by GV, supervised by DG.

Quantitative and qualitative data were, after the above mentioned analyses, interpreted together in relation to the research questions.

#### Quality of interviews

GV reflected on the course of the interview, the agreement between the interviewees, the impression of the interviewees, the first impression of the added value of the interview, and whether there were moments of being suggestive after every interview [40]. GV wrote memos during data collection and analysis. GV and ML wrote memos during the interview analysis in a shared document. After six interviews, they decided to elaborate on the topics of non-pharmacological interventions, physical restraints and psychotropic drugs as they often lacked in-depth information concerning why these were applied in treatment. We followed the consolidated criteria for reporting qualitative research (COREQ) for the qualitative parts (see Supplementary Material 5 [42]).

### Ethics statement

The study was conducted in accordance with the Declaration of Helsinki as well as the rules applicable in the Netherlands. The local Medical Ethics Review Committee, CMO region Arnhem-Nijmegen at the Radboud University Medical Center, stated that the Medical Research Involving Human Subjects Act (WMO) does not apply to this study and that an official approval of this study was not required (reference number 2018–4354). Informed consent was obtained from all participants, i.e. unit managers and interviewees, prior to data collection.

## Results

### Organizational characteristics (research question 1)

#### Admission and discharge characteristics

The majority of patients were admitted from regular DSCUs, home or a mental health care institution. Details of the admission and discharge characteristics per unit can be found in Supplementary Material 1. Before admission, the admission criteria were checked in terms of severe challenging behavior and (suspected) dementia. In three units, a maximum of two to three patients with physical aggression was allowed. Nine units exclusively treated patients with dementia, whereas three units also treated other older patients within the same or another sub-unit. Psychiatric comorbidity was not an exclusion criterion, except alcohol dependency in three units, and reflected the rule rather than the exception according to unit managers and interviewees. The proportion of compulsory admissions on a unit varied between 4 and 90% (median 20%) at the moment the digital questionnaire was completed. The mean age of patients ranged from 65 to 82 years. At admission, the vast majority of patients used many different types of psychotropic drugs, often without a good rationale, according to the interviewees. Some interviewees mentioned that the severe challenging behavior for which patients were admitted was not present after admission in a few cases, and suggested that another social and/or physical environment may explain this. The length of stay ranged from one to twelve months. The majority of patients were discharged to regular DSCUs, and the proportion of deaths ranged between 6 and 63% (median 19%) on average per unit per year.

#### Staffing

A multidisciplinary team comprising at least a physician responsible for medical care, a psychologist and a nursing staff member but often more professionals such as therapists treated the patients. One, two or three physicians were responsible for medical care (see Supplementary 2): eleven elderly care physicians, two geriatricians, and three (geriatric) psychiatrists. In six units, (geriatric) psychiatrists were permanently involved in the treatment. In four units, a psychiatrist was sometimes consulted. Psychiatrists were valued by the interviewees for their expertise regarding the prescription of psychotropic drugs and psychiatric diagnostics. In three units, neurologists were permanently involved for their expertise in diagnostic problems in neurodegenerative diseases. In all units, therapists such as physiotherapists, occupational therapists speech therapists and dieticians were involved by the physician when necessary. A few units had a music therapist or psychomotor therapist involved. One unit had therapists who had received extra training in sensory integration [43]. This unit also employed personnel who were so-called miMakkus clowns, which is a practice-based psychosocial intervention using clowning for people with advanced stage dementia with the goal to make contact where communication in the usual cognitive way is no longer possible [44].

In ten units, baccalaureate-educated registered nurses worked in relatively low numbers, but the vast majority comprised registered vocationally trained nurses, and certified nurse assistants with a median average age of 38 years. Most units had a vast majority of registered vocationally trained nurses (*n* = 5) or a vast majority of certified nurse assistants (*n* = 5). The availability of nursing staff hours per patient substantially differed among units, ranging from 2.9 to 6.2 nursing staff hours per 24 h per patient (median 3.9). The median average sick leave was 5% in the former year (without maternity leave). All but one unit had vacancies for nursing staff (details per unit can be found in Supplementary Material 3). A stable team was seen as important, and thus in some units nursing staff were employed for a minimum of 24–32 contract hours per week.

Nursing staff were seen as central in the treatment by the interviewees: *“They [nursing staff members] also try things before agreements [about management] are made. They are often the ones who come up with new approaches. We also come up with them, but I think that the performing and also coming up with is a very big task of the [nursing staff] team” (unit 11).* Competences that were seen as important in nursing staff by both unit managers and interviewees included being open to new approaches, flexibility, reflectiveness on one’s own behavior, being good at observing and describing behavior, and being able to cope with stressful situations such as aggression. The ability to provide personal care with a caring attitude together with being able to set boundaries and act upon the challenging behavior was seen as key: *“On the one hand, you should be able to provide warm personal care and be creative, but you should also be able to be directive when necessary and sense when you should approach someone from below and when from above” (unit 03).* At one unit, the staff were also trained in the principles of miMakkus for communication in alternative ways [44]. Most units provided training for nursing staff to manage with physical aggression. Some units started with peer consultation focusing on the experience of caring for patients with severe challenging behavior, led by the psychologist. It helped staff in being able to set boundaries and gaining confidence in their ability to search for and apply suitable interventions. The support of the nursing staff manager was seen as important. Attention to work balance, mental support and extra staff during times of crisis helped in preventing sick leave and being more open to new behavioral approaches, according to the interviewees.

Six units involved volunteers, whereas in the other units interviewees considered this impossible due to the severity of the patients’ behavior. Units with volunteers focused on recruiting volunteers who could manage severe challenging behavior, and strongly invested in their supervision.

#### Physical environment

##### General impression

Unit sizes ranged from 10 to 28 one person rooms available. Three units had the possibility to walk all around the unit. Interviewees mentioned their experience that this could reduce agitation in some patients and was missed when not available. Three units had sub-units with very low visual stimuli and very few objects. One sub-unit had only very soft objects. Eight units had seclusion rooms and in eight units enclosure beds were available, namely a bed with a canopy with zippered panels attached to a height-adjustable bed [45, 46]. Details of the general impression per unit can be found in Supplementary Material 6.

##### OAZIS-dementia

The theme of safety scored highest on average in terms of the probability that the environment has a positive effect on the safety of a patient, while the theme of domesticity scored lowest (Table 3). Other individual items that scored relatively low were reducing noise by spatial planning (*n* = 11), bath rooms not being visible from the general living room (*n* = 6), and bath rooms not being directly accessible from the patients’ room (*n* = 7). The unit with the lowest score (unit 10) had a low score on the view and nature, and invested little in domesticity. The unit with the highest score (unit 01) had invested in the physical environment of the unit with special attention to sensory stimulation.

**Table 3:** OAZIS-dementia^a^

	Privacy and autonomy	Sensory stimulation	View and nature	Facilities	Orientation and routing	Domesticity	Safety	Total
*Unit no*	Item no. 1–7	Item no. 8–25	Item no. 26–36	Item no. 37–45	Item no. 46–52	Item no. 53–69	Item no.70–72	Item no. 1–72
*01*	4.6	4.5	4.1	4.3	4.5	3.6	5	4.2
*02*	4.3	3.7	3.5	3.8	3.6	3.2	4.7	3.6
*03*	4.3	4.4	3.7	4.4	4	3.5	3	4
*04*	4.3	3.8	3.5	3.4	3.4	3.9	5	3.8
*05*	3.7	3.8	3.8	4.2	3.3	3.5	5	3.8
*06*	3.4	3.8	3.4	3	3.6	3.1	4.3	3.4
*07*	4.9	3.5	3	3.1	3.7	3.5	5	3.6
*08*	4.4	4.3	3	4.2	4.4	3.9	4.7	4
*09*	3.9	3.9	3.4	3.1	3.4	3.5	5	3.6
*10*	4.1	3.4	2.5	3.1	3.9	2.9	5	3.3
*11*	4.1	3.8	4.2	4.2	3.9	3.6	4.7	3.9
*12*	4.1	4.1	3.4	Missing	3.9	2.8	4	3.7
*13*	4.7	Missing	3.8	3.8	3.7	3.4	4.3	4
Average per theme	4.2	3.9	3.5	3.7	3.8	3.4	4.6	3.8

^a^Averages of the themes of the OAZIS-dementia: averages per theme per unit, theme (last row), and unit (last column). Averages are calculated back to the range on a scale from 1 ‘not at all’ to 5 ‘completely applicable’. Higher scores indicate a higher probability of the environment having a positive effect on its residents [37]

### Management (research question 2)

Units varied in the degree in which they used a more intuitive or methodological work-up. Two interviewed elderly care physicians described their work-up explicitly as intuitive, although this was nuanced in one interview by the psychologist. In three units, specific evidence-based methods and/or multi-disciplinary programs developed for regular DSCUs were used, such as the ABC method, and the multidisciplinary programs STA-OP! protocol and Grip on Challenging Behavior [47–49]. Three units had explicit wishes or plans for training in a multidisciplinary program.

#### Before admission

Prior to admission, it was considered critically whether treatment was needed. Units considered which interventions had been used to date, and often gave advice to prevent admission. In one unit, the interviewee mentioned they insisted on consultation in the current residence beforehand, thereby preventing about one-third of proposed admissions. This prior consultation was conducted by the physician responsible for medical care or the psychologist, sometimes together with a nursing staff member. In three units, there was close collaboration with an ambulant team within the organization that advised in home situations.

#### Diagnostics

Interviewees explained that they had a program of clinical investigation in the first week after admission, comprising an analysis of the medical history in conjunction with the (psychotropic) drug use, physical and psychiatric examination, laboratory examination, making a first plan for the behavioral approach with interventions for the nursing staff, and a hetero-anamnesis of the biography, often with attention to personality and coping style. Two interviewees mentioned that without a biography it was often difficult to treat these patients well: *“Yes, that’s when you miss quite a part of the puzzle. This can make it very difficult to draw conclusions,. in which case you find yourself struggling to find the correct approach for quite some time” (unit 04).* All units paid attention to sensitivity for sensory stimuli, although the intensity and expertise available differed among units. Tolerance of a certain level of challenging behavior was essential in this phase to enable effective observation: *“If someone wants it [the challenging behavior] gone immediately, it changes your perspective. There’s a certain peace like: ‘okay, this is it, let’s see where we still can be of any help to someone’” (unit 05)*. *“A very enthusiastic team that is really able to let people be. I find that really important too. [A team] that does not react immediately but is able to let it run its course for a while and see what happens together” (unit 11).* The multidisciplinary team interpreted the behavior and discussed treatment every week (every other week in one unit). To ensure a consistent approach by the nursing staff, attention to differences in the experience and interpretation of the behavior was seen as essential.

#### Treatment

For most patients, the treatment comprised a combination of non-pharmacological interventions and psychotropic drugs. Although interviewees strived to taper off the psychotropic drugs, they did not always consider this to be possible. They were satisfied when they could reduce the number of different types of psychotropic drugs and prescribe psychotropic drugs with a better rationale. Overall, interviewees mentioned that guidelines held limited usefulness for the treatment in these units: *“Almost everything we do is no longer evidence-based and that’s a huge problem.” “We all have mainly expert opinions, meaning the knowledge of people who know more about it” (unit 06).* Interviewed psychiatrists described that they used the psychiatric guidelines more freely than usual: *“For example, in severe disinhibited behavior—not sleeping any more, being very restless. You can also interpret this as manic and we treat it as manic, and we find we achieve good results. We try especially try to find which box to tick, because the guideline is not able [to provide for a proper label], which label fits best and try to treat for that” (unit 02).* As a clinical evaluation instrument of the challenging behavior, four units completed the Neuropsychiatric Inventory (NPI-Q) and the Cohen-Mansfield Agitation Inventory (CMAI) at regular intervals [50, 51]. In two units, these were used in the actual evaluation of the treatment. In one of these units, goals were identified and evaluated with a goal attainment score.

Overall, visual stimuli were minimized and few objects were available to prevent over-stimulation and harm. In three units, patients were first admitted to a sub-unit with very few stimuli, before being moved to a sub-unit with more stimuli when they showed less aggression. Enclosure beds were also used to reduce stimuli, but also for improving sleep during the night, reducing ongoing restlessness and preventing falls. Other examples of specific interventions in sensory stimuli were deep pressure through a weighted vest or a headphone.

Non-pharmacological interventions used varied among the units, and included video-interaction training, sensory integration therapy, music therapy, Snoezelen, psychomotor therapy, and principles of “powerless in daily living” (PDL) care, a type of emotion-oriented care for patients with an irreversible self-care deficit [52]. As previously mentioned, one unit also used the principles of miMakkus, one unit paid special attention to the role of sleeping disorders, and one stimulated a break with patterns in the family system by discouraging visits during the first two weeks after admission. In one unit, patients with therapy-resistant severe challenging behavior were sometimes treated with electroconvulsive therapy with relevant results, although the therapy had to be continued to sustain the results.

#### Discharge

Discharge was regarded possible when the patient’s behavior was expected to be manageable in a regular DSCU. Discharge was often difficult due to the specific needs of the patients, while being stigmatized by the assumed psychiatric comorbidity of potential units was also a problem according to interviewees from units with a background in mental health care. Some interviewees mentioned that discharge seemed to be impossible for some patients, sometimes after a probation discharge: *“I might say that we go on trying, but that’s actually not always the case. Because at a certain moment we simply don’t know any more, than it’s manageable for the unit.” “Exactly, sometimes it’s manageable for us, and then we say that this is the best possible. But we mean that it’s not manageable in a regular unit” (unit 05).*

Some units strongly invested in discharge by inviting the nursing staff of the proposed unit for discharge to care for the patient together to explain behavioral guidance in practice. These units’ teams were also available for the new units after discharge.

## Discussion

The main finding of this study is that units are pioneering and have strong heterogeneity in the management of severe challenging behavior in dementia. This heterogeneity was demonstrated by the varying degree to which a more intuitive or methodological work-up was used, the broad variety of non-pharmacological interventions used, and the differences in nursing staff hours, nursing staff education levels, length of stay, and the physical environment. Despite these differences, there were similarities in emphasis on observation with an open attitude, the key role of nursing staff, frequent multidisciplinary meetings, and attention to sensory stimuli.

### Management

Although units varied in the degree to which they adopted a more intuitive or methodological work-up and the fact that a broad variety of non-pharmacological interventions was used, the ability – especially of the nursing staff members – to observe behavior was seen as key. These observations together with an analysis of the (non-)medical biography and personality were interpreted and discussed by management in the multidisciplinary team meetings. From literature, we know that pre-morbid personality may play a role in challenging behavior [53]. In a qualitative study in patients with extreme challenging behavior in regular DSCUs, sub-optimal interdisciplinary collaboration and communication was one of the factors that contributed to the experience of an impasse [54]. The frequent multidisciplinary meetings may have facilitated collaboration and communication, although from our own research about severe challenging behavior we also know that this needs to be facilitated by process conditions such as the organization’s support of the professionals, and clear agreements and defined roles [55].

All units paid attention to sensory stimuli that were thought to affect the behavior, although the methods to analyze this and their intensity varied among units. In some units, special adaptions to the physical environment were made. Challenging behavior may be due to sensory impairment and/or sensory processing abnormalities [56, 57], which therefore require assessment and individualized sensory stimuli. Compulsory admissions were common, which means that the challenging behavior caused danger to oneself or others [58]. These and other possible coercive measurements in the form of physical restraining interventions such as enclosure beds and seclusion rooms were used to prevent harm or diminish sensory stimuli. However, further research into the effectiveness of interventions that are or may be physically restraining is necessary.

### Role of nursing staff

The nursing hours per patient per 24 h substantially differed among units. The median average of 3.9 h per patient per day is similar to the current hours per resident per day in regular nursing home units in the United States [59]. Despite this, in five units the education level of nursing staff was higher than in regular DSCUs in the Netherlands, and all units hired nursing staff with specific competences. Nursing staff competences that were seen as relevant were an openness to new approaches, flexibility, reflectiveness, being able to observe behavior well with a certain tolerance towards challenging behavior, and being able to cope with stressful situations. Indeed, these are competences that are known to be important in regular dementia care [60–62]. Moreover, a consistent approach by the nursing team seems essential, which was facilitated discussing the interpretations of the behavior. A consistent approach by the nursing staff and an open attitude of those involved in the direct environment have also been found to be part of the successful treatment of severe challenging behavior [55].

Being open to new approaches, showing a certain tolerance towards the behavior, and coping with stressful situations possibly corresponds with the competence of the therapeutic use of self, which includes perseverance, situational awareness, and the ability to be present [61]. This therapeutic use of self probably requires a reflectiveness on one’s own behavior as a nursing staff member. Learning this is part of training as a registered nurse, but not as a nursing assistant [63, 64]. The participating units fostered this reflectiveness on one’s own behavior by recruiting nursing staff, and some units offered training through peer consultation. This reflectiveness may also be valuable in and improved by the frequent multidisciplinary meetings.

#### Strengths and limitations

There are two main strengths of this study. First, the integration of different types of data collection offers rich insights into the organization of these units. Second, this study represents the organization and management of challenging behavior of highly specialized units in the Netherlands, with thirteen out of sixteen known units having participated.

There are some possible limitations to this study. First, the data were collected in 2018 when several of these pioneering units had recently started. Therefore, characteristics and management of behavior on these units may have developed, and insights may have changed from the experience of these pioneering units. Second, we found that most unit managers did not have complete data, which is a concern for better monitoring in the future. Moreover, this led to estimations by the unit managers and therefore led to less precise data. Third, we asked the physician responsible for medical care to invite another practitioner – such as the psychologist – whom he/she considered important in the treatment. Nurses and nursing assistants were not interviewed about the experiences and competences that they consider useful in their work, which may have led to selection bias towards the perspective of what is relevant for the physician. Moreover, nursing staff was considered as most important in the management of challenging behavior, meaning that their perspective is particularly relevant and that further research should include this. Fourth, interventions used in the management of challenging behavior may have remained unmentioned, whereby data saturation was not reached concerning this. Despite this, the main finding of heterogeneity in interventions persists together with the representativeness for the Netherlands. Fifth, the units differed in their experience and expertise, i.e. six units had opened less than two years prior to the study, which may have resulted in less in-depth interviews.

## Conclusions and implications

We found that these pioneering units have strong heterogeneity in their organization and management of severe challenging behavior in people with dementia. This finding emphasizes the need for further research into what is effective in interventions, the (social) context such as the attitude of persons surrounding the patients, as well as the physical environment. The framework for complex interventions may prove useful to investigate this [65]. Furthermore, research into the necessity of these highly specialized units could shed light on what is needed on regular DSCUs to manage challenging behavior better and prevent transfers of patients. Recent research in patients admitted to some of these highly specialized units has shown that increasing severity of the challenging behavior, realization that the needs of the person with dementia cannot be met, and the burden of the nursing staff—often triggered by a life-threatening event—may lead to these admissions [66]. Combining this knowledge with information about organizational influences on both highly specialized units and DSCUs, such as already known influences, i.e. staff availability, staff training, the use of specific methods such as dementia care mapping, and influence of the physical environment [15, 16, 26, 27, 67], but also societal developments such as the tendency to live at home longer [68], could provide relevant insights for improving the quality of care on both DSCUs and highly specialized units. This also holds for insight into specific patient characteristics of patients admitted to highly specialized units such as dementia type, character and severity of the challenging behavior, and whether and why treatment is effective.

Although this study found a great variety in organization and management of severe challenging behavior, we think that three suggestions for practice can be formulated. First, nursing staff plays a key role in the management of the behavior. A stable, higher educated team with many contract hours per nursing staff member as well as a certain tolerance for severe challenging behavior to observe well was described as necessary. Second, investing in the physical environment seems to be of value. Safety, a low amount of visual and auditive stimuli, space and interventions to dose stimuli individually probably add to the wellbeing of patients on these units. Third, the involvement of expertise from mental health care was valued. These possible implications deserve further study.

### Supplementary Information

Supplementary Material 1.

Supplementary Material 2.

Supplementary Material 3.

Supplementary Material 4.

Supplementary Material 5.

Supplementary Material 6.

## Data Availability

Data from the digital questionnaires are available in Supplementary Materials 1, 3 and 6. Data of the interviews are not publicly available to ensure the interviewees’ privacy, but are available on reasonable request from the corresponding author (gerrie.vanvoorden@radboudumc.nl). Data from the observation of the physical environment are available on request from the corresponding author.
