# Supplementary material for: Organizational characteristics of highly specialized units for people with dementia and severe challenging behavior
Source: BMC Geriatr. 2024 Aug 14;24:681. doi: 10.1186/s12877-024-05257-x (PMC11323444; doi:10.1186/s12877-024-05257-x)
Supplement: Supplementary file 1 — Supplementary Material 1. [file 12877_2024_5257_MOESM1_ESM.docx]

| **Supplementary 1: Patient characteristics per unit** | | | | | | |
| --- | --- | --- | --- | --- | --- | --- |
| ***Unit*** | *Residence before admission** | *Compulsory admission** | *Average age patients (years)* | *Average length of stay (months)* | *Discharge location** | *Death rate* |
| **01** | - home 72%  - long term dementia care 13%  - hospital 9%  - mental health care 3%  - other: care for mentally disabled 3% | 10% | 79 | 3 | - home 32%  - back to referring unit 1%  - long term care unit in long term care organization 66%  - mental health unit for long term care 1% | 12% |
| **02** | - home 7%  - long term dementia care 41%  - hospital 7%  - mental health care 45% | 67% | 73 | 5 | - home 5%  - back to referring unit 10%  - long term care unit within organization 45%  - long term care unit in long term care organization 40% | 22% |
| **03** | - home 31%  - long term dementia care 6%  - hospital 2%  - mental health care 61% | 62% | 72 | 6 | - home 26%  - long term care unit in long term care organization 50%  - other 24%: other in treatment in general mental health care 15%, hospital 9% | 6% |
| **04** | - home 29%  - long term dementia care 58%  - long term somatic care 2%  - mental health care 9%  - other 3%: unit for acquired brain injury, residential care home | 20% | 74 | 7 | - home 6%  - back to referring unit 24%  - long term care unit within organization 42%  - long term care unit in long term care organization 11%  - mental health unit for long term care 1%  - no discharge (patient stays) 15% | 29% |
| **05** | - home 36%  - long term dementia care 56%  - mental health care 8% | 37% | 73 | 6 | - back to referring unit 3%  - long term care unit within organization 51%  - long term care unit in long term care organization 17%  - no discharge (patient stays) 30% | 23% |
| **06** | - home 25%  - long term dementia care 55%  - hospital 10%  - mental health care 10% | 80% | 70 | 4 | - home 5%  - back to referring unit 60%  - long term care unit within organization 5%  - long term care unit in long term care organization 30% | 8% |
| **07** | - home 8%  - long term dementia care 34%  - long term somatic care 17%  - hospital 8%  - mental health care 17% | 4% | 76 | 12 | - home 20%  - long term care unit within organization 43%  - long term care unit in long term care organization 30%  - mental health unit for long term care 8% | 20% |
| **08** | - home 42%  - long term dementia care 17%  - long term somatic care 17%  - hospital 8%  - mental health care 17% | 10% | 65 | 8 | Insufficient data available because unit started since several months, discharges were to:  - long term care unit within organization  - long term care unit in long term care organization | 63% |
| **09** | - home 65%  - long term dementia care 10%  - long term somatic care 10%  - hospital 10%  - mental health care 5% | 20% | 76 | 4 | - back to referring unit 10%  - long term care unit within organization 46%  - long term care unit in long term care organization 4%  - no discharge (patient stays) 40% | 50% |
| **10** | - home 10%  - long term dementia care 70%  - hospital 20% | 20% | 75 | 5 | - back to referring unit 60%  - long term care unit within organization 20%  - long term care unit in long term care organization 20% | 18% |
| **11** | - home 5%  - long term dementia care 20%  - hospital 10%  - mental health care 65% (35% from a mental health care unit in the hospital) | 20% | 81 | 2 | - back to referring unit 68%**  - long term care unit within organization 4%  - long term care unit in long term care organization 3%  - other 25%: hospital 4%, unknown 23% | 8% |
| **12** | - home 23%  - long term dementia care 10%  - hospital 11% mental health care 2%  - other 54%: unknown | 38% | 82 | 1 | - home 24%  - back to referring unit 10%  - long term care unit within organization 7%  - long term care unit in long term care organization 56%  - mental health unit for long term care 3% | 3% |
| **13** | - home 75%  - long term dementia care 10%  - hospital 5%  - mental health care 10% | 90% | 80 | 3 | - home 13%  - long term care unit within organization 17%  - long term care unit in long term care organization 68%  - no discharge (patient stays) 1% | 24% |
| * These results are probably not precise due to estimation.  ** When admitted from hospital or mental health care to unit 11 this meant the place before (no data available about this). | | | | | | |
