# Supplementary material for: Organizational characteristics of highly specialized units for people with dementia and severe challenging behavior
Source: BMC Geriatr. 2024 Aug 14;24:681. doi: 10.1186/s12877-024-05257-x (PMC11323444; doi:10.1186/s12877-024-05257-x)
Supplement: Supplementary file 2 — Supplementary Material 2. [file 12877_2024_5257_MOESM2_ESM.docx]

| **Supplementary 2: Involvement of physicians** | | | | | |
| --- | --- | --- | --- | --- | --- |
| **ID (background)** | **Physician responsible for medical care 1** | **Physician responsible for medical care 2** | **Physician responsible for medical care 3** | **Consultant psychiatrist** | **Other consulting physician(s)**** |
| **01 (mental health care)** | elderly care physician* | psychiatrist ^~ |  | Not applicable | Sometimes consults neurologist |
| **02 (mental health care)** | geriatric psychiatrist * | elderly care physician |  | Not applicable | Sometimes consult neurologist, rehabilitation doctor, geriatrician |
| **03 (mental health care)** | elderly care physician* | geriatric psychiatrist |  | Not applicable | Neurologist |
| **04 (long term care)** | elderly care physician* |  |  | Yes, psychiatrist (specialization unknown) sees every patient once in four weeks |  |
| **05 (long term care)** | elderly care physician* |  |  | Yes, psychiatrist (specialization unknown) involved in at least every multidisciplinary meeting |  |
| **06 (collaboration of mental health care and long term care)** | geriatrician* | psychiatrist***◦** | elderly care physician | Not applicable |  |
| **07 (long term care)** | elderly care physician* |  |  | Occasionally this physician discusses patients by phone |  |
| **08 and 09 (long term care)** | elderly care physician* |  |  | Occasionally this physician discusses patients by phone | Sometimes consults neurologist |
| **10 (long term care)** | elderly care physician* |  |  | Yes, psychiatrist (specialization unknown) sees patients every six weeks, in collaboration with elderly care physician who works in the consulting mental health care organization, this physician sees patient every two weeks |  |
| **11 (long term care)** | elderly care physician* |  |  | Occasionally this physician discusses patients by phone |  |
| **12 (mental health care)** | geriatrician* | geriatric psychiatrist^ |  | Not applicable | Neurologist |
| **13 (mental health care)** | elderly care physician* | psychiatrist^~ |  | Not applicable | Neurologist (4 contract hours a week), sees every patient |
| * Was also interviewed.  ** This is information from interviews and may be incomplete concerning the occasional calls to specialists.  ^ In this unit this was only a formality due to the criteria of the insurance. These psychiatrists were involved in the treatment of all patients, but in practice not in the role of the physician responsible for medical care.  ~ Uncertain whether this was a geriatric or general psychiatrist.  **◦** In unit 06 normally a geriatric psychiatrist worked as responsible physician, but he was replaced temporarily by a general psychiatrist. | | | | | |
