# Supplementary material for: Organizational characteristics of highly specialized units for people with dementia and severe challenging behavior
Source: BMC Geriatr. 2024 Aug 14;24:681. doi: 10.1186/s12877-024-05257-x (PMC11323444; doi:10.1186/s12877-024-05257-x)
Supplement: Supplementary file 3 — Supplementary Material 3. [file 12877_2024_5257_MOESM3_ESM.docx]

| **Supplementary 3: Nursing staff characteristics per unit** | | | | | | |
| --- | --- | --- | --- | --- | --- | --- |
| ***Unit*** | *Unit size* | *Nursing staff*  *(in full time equivalents)* ^* | *Nursing hours/24 hours/patient*  *(staff-patient ratio)* | *Sick leave nursing staff*  *(%)* | *Average age nursing staff (years)* | *Vacancies nursing staff* |
| **01** | 17 | **- EQF 3: 12.28**  - EQF 4: 1  - EQF 6: 2.11 | 4.4 | 1.2 | 35 | 1 |
| **02** | 28 | - EQF 2: 3  **- EQF 3: 9**  **- EQF 4: 8**  - EQF 6: 2 | 3.2 | 8 | 35 | 1 |
| **03** | 21 | - EQF 2: 1.5  - EQF 3: 1.5  **- EQF 4: 11**  - EQF 6: 3 | 3.5 | 5 | 38 | 0 |
| **04** | 12 | - EQF 2: 0.67  - EQF 3: **5.42**  - other: ‘care companion’ 0.1 | 2.9 | 5 | 35 | 1 |
| **05** | 24 | - EQF 2: 3.14  - **EQF 3: 12.16**  - EQF 4: 3.52  - EQF 6: 0.88 | 3.6 | 2.7 | 40 | 1 |
| **06** | 19 | - EQF 2: 1  - EQF 3: 4  **- EQF 4: 15**  - EQF 6: 2 | 4.1 | 8 | 35 | 1 |
| **07** | 25 | - EQF 2: 1.5  **- EQF 3: 5.4**  **- EQF 4: 4.3**  - EQF 6: 0.67 | 3.9 | 7 | 40 | 6 |
| **08** | 12 | - EQF 2: 1  - EQF 3: 2  **- EQF 4: 5**  - EQF 6: 1 | 3.8 | 0.6 | 33 | 1 |
| **09** | 10 | **- EQF 3: 8.89**  - EQF 4: 1.76  - EQF 6: 1 | 3.8 | 5 | 35 | 0 |
| **10** | 11 | **- EQF 3: 4**  **- EQF 4: 6** | 4.3 | 3 | 45 | 1 |
| **11** | 10 | - **EQF 3: 8.35**  - EQF 4: 2.56  - EQF 6: 1.78  - other:  miMakkus-clown: 0.3 | 6.2 | 9.5 | 43 | 1 |
| **12** | 16 | - EQF 2: 0.78  - EQF 3: 3.11  - **EQF 4: 13.86** | 5.1 | 6 | 47 | 2 |
| **13** | 17 | - EQF 3: 1.56  - **EQF 4: 11.04**  - EQF 6: 2.56 | 4.2 | 12 | 40 | 1 |
| * For most units this numbers did not match the separately asked total of fulltime equivalents (including vacancies) and/or precise numbers could not be obtained.  ^ Fulltime is calculated as 36 contract hours a week. | | | | | | |
