# Supplementary material for: Organizational characteristics of highly specialized units for people with dementia and severe challenging behavior
Source: BMC Geriatr. 2024 Aug 14;24:681. doi: 10.1186/s12877-024-05257-x (PMC11323444; doi:10.1186/s12877-024-05257-x)
Supplement: Supplementary file 4 — Supplementary Material 4. [file 12877_2024_5257_MOESM4_ESM.docx]

| **Supplementary 4. Interview guide of interview with physician responsible for medical care and another practitioner** | |
| --- | --- |
| ***Topics:*** | *Questions:* |
| *Before admission* | For the period from registration for admission to admission: Who are involved? How is the collaboration with other care providers? |
| *First day on the unit* | What is the process of a new admission on the first day on the unit? |
| *Admission after first day* | What is an admission like after this?  - What does a day on the unit look like?  - Who are involved during the course of an admission? How? Who do you consult? When? Do they see the person with dementia in person? What is your experience in the collaboration with mental health care/nursing home institution (choose what is applicable)?  - What guidelines and/or methods are used? What is your experience with these?  - What is the role of non-pharmacological interventions in the treatment of severe challenging behavior?*  - How do you see the role of the nursing team? What does the collaboration look like? What characterizes a nurse or nursing assistant who fits in this unit?  - How are family or other informal caregivers involved?  - Ask through about methodological or intuitive characterized work-up.*  - Ask through about physical restraints.*  - Ask through about psychotropic drugs used. How often? What? Experience with this?* |
| *Explanation length of stay longer* | *If the expected and actual lengths of stay differ:* What explains the difference between the expected and actual durations? |
| *Characterization treatment* | What characterizes your approach in treating patients with severe challenging behavior? What makes this unit different from the work-up at home or in a nursing home?* |
| *Evaluation treatment* | How does evaluation of treatment take place? Who are involved? Is there a set format? To what extent do/does the treatment vision/protocol/method support this? |
| *Training management behavior* | How much room is there for training of staff in managing severe challenging behavior? What does this training look like? |
| *Training specific methods* | What attention is paid in the training about methods used in this unit for the treatment of challenging behavior? What does this training look like? |
| *Satisfied with treatment* | When are you satisfied with the treatment? How did you accomplish such a result? |
| *Experienced difficulties* | There will be times when treatment does not work out as hoped for. What are issues that you face? How do you deal with these? |
| *Completion* | Summarize and check. Are the other issues you would like to share? |
| ** Added after six interviews.* | |
