# Supplementary material for: Organizational characteristics of highly specialized units for people with dementia and severe challenging behavior
Source: BMC Geriatr. 2024 Aug 14;24:681. doi: 10.1186/s12877-024-05257-x (PMC11323444; doi:10.1186/s12877-024-05257-x)
Supplement: Supplementary file 6 — Supplementary Material 6. [file 12877_2024_5257_MOESM6_ESM.docx]

| **Supplementary 6: start year and general information physical environment per unit** | | | | | |
| --- | --- | --- | --- | --- | --- |
| ***Unit*** | *Start year* | *Unit size* | *Subunits*  *(Specific subgroups and/or other indication)* | *Seclusion room(s)* | *Enclosure bed* |
| **01** | 2004 | 17 | No subunits.  (Other indication: patient group admitted for cognitive diagnostics often with mild challenging behavior.) | Yes | No |
| **02** | 2017 | 28 | Two subunits, physically closed to each other.  (No difference in patient group for the subunits.) | Yes | Yes |
| **03** | 2014 | 21 | Two subunits, possible to close them to each other, normally open.  (Other indication: one subunit for patient group with primarily psychiatric diagnosis and cognitive problems.) | Yes | Yes |
| **04** | 2013 | 12 | Two subunits, physically closed to each other.  (One subunit for very severe behavioral problems.*) | No | Yes |
| **05** | 2016 | 24 | Four subunits, one subunit mostly closed to the other three units and possible to close them all to each other.  (One subunit for very severe behavioral problems* with a maximum of three patients with physical aggression.) | Yes | Yes |
| **06** | 2016 | 19 | Two subunits, physically close to each other.  (One subunit for very severe behavioral problems*. Other indication: Incidentally patients with a primarily psychiatric diagnosis.) | Yes | No |
| **07** | 2009 | 25 | Three subunits, physically closed to each other.  (Other indication: one subunit for elderly with primarily psychiatric diagnosis.) | No | Yes |
| **08** | 2017 | 12 | No subunits.  (No subgroups or other indications.) | No | No |
| **09** | 2014 | 10 | No subunits.  (No subgroups or other indications.) | Yes | Yes |
| **10** | 2010 | 11 | No subunits.  (No subgroups or other indications. A maximum of three patients with physical aggression.) | Yes | No |
| **11** | 2012 | 10 | No subunits.  (No subgroups or other indications.) | No | Yes |
| **12** | 1977 | 16 | No subunits.  (No subgroups or other indications.) | No | Yes |
| **13** | 1994 | 17 | No subunits.  (No subgroups or other indications.) | Yes | No |
| * Patients are admitted to the other subunit(s) when the challenging behavior, often physical aggression, declines. | | | | | |
